# Prevalence and determinants of disability among adults in Malaysia: results from the National Health and Morbidity Survey (NHMS) 2015

**DOI:** 10.1186/s12889-017-4793-7

**Published:** 2017-09-29

**Authors:** Noor Ani Ahmad, Noraida Mohamad Kasim, Nur Azna Mahmud, Yusniza Mohd Yusof, Salimah Othman, Ying Ying Chan, Mohamad Aznuddin Abd Razak, Muslimah Yusof, Maisarah Omar, Fazly Azry Abdul Aziz, Rasidah Jamaluddin, Norazizah Ibrahim Wong, Tahir Aris

**Affiliations:** 1 Centre for Family Health Research, Institute for Public Health, Ministry of Health, Jln Bangsar, 50590 Kuala Lumpur, Malaysia; 2 Cheras Rehabilitation Hospital, Ministry of Health, Bandar Tun Razak, 56000 Kuala Lumpur, Malaysia; 3 Family Health and Development Division, Ministry of Health Malaysia, Kompleks E, Pusat Pentadbiran Kerajaan Persekutuan, 62590 Putrajaya, Malaysia

**Keywords:** Disability, Washington group, Population-based survey, Malaysia

## Abstract

**Background:**

Reliable national data on disability which is internationally comparable is needed in Malaysia. This study aims to examine the prevalence of disability among adults in Malaysia and its determinants, particularly the socioeconomic factors and comorbidities.

**Methods:**

This study was based on the disability module, which is part of the National Health and Morbidity Survey 2015. This survey was implemented using a multi-stage stratified sampling design. A locally validated Washington Group questionnaire was used to collect data on disability.

**Results:**

Based on the definition of having at least one domain scored "a lot of difficulty or unable to do at all" or at least “some difficulty” scored in two domains, the prevalence of disability among adults in Malaysia was 11.8% (95% CI: 11.15, 12.53). Logistic regression analysis performed showed that population at risk of having disability in Malaysia were those of older people, ethnic minority, low level of education, single, obese, physically inactive and having mental health problems. Among older people, disability was significantly higher among those with no formal education, having mental health problems and physically inactive.

**Conclusions:**

The prevalence of disability among adults in Malaysia is comparable to WHO estimates and most developing countries. Planning for healthcare services should consider at-risk population, particularly older people and those from disadvantaged background to ensure equity healthcare.

## Background

International Classification of Functioning, Disability and Health (ICF) described disability as an umbrella term, encompassing impairment, activity limitations, and participation restrictions [1]. The UN Convention on the Rights of Persons with Disabilities emphasis that “all persons with all types of disabilities should enjoy every human right and fundamental freedom”, while Objective 3 of the World Health Organization (WHO) Global Disabilities Action Plan, 2014–2021 urged each member states “to strengthen collection of relevant and internationally comparable data on disability and support research on disability” [2]. As such, reliable data on disability and its association with other comorbidities are required for the planning of disabilities inclusive policies, programmes and strategies in Malaysia.

Approaches in measuring disability vary across countries and may influence the results. The measures vary according to the purpose and application of the data, the aspects of disability examined; the definitions, question design, reporting sources, data collection methods, and expectations of functioning. Thus, the prevalence also varies from as low as 0.7% in Kenya to 20.0% in Australia [3], while WHO estimates 15% of the world’s population have any disability [4]. In addition, Mitra et al. estimated the global prevalence of adults with disability at 14% based on the analysis of the World Health Survey (WHS) (2002–2004) data [5].

The international standard to describe and measure health and disability is by using ICF, which was officially endorsed by all 191 WHO Member States in the 54th World Health Assembly on 22 May 2001 [5]. However, the ICF is very lengthy due to its comprehensiveness and was considered not feasible to be included in the regular health survey conducted by most countries [6]. Therefore, the development of short set questionnaire based on ICF framework by the Washington Group on Disability Statistics (WG) was very much welcome. This questionnaire was intended to be used in census methodology for producing internationally comparable data. The WG short set question aims to identify people in the population who are at greater risk than the general population of experiencing limited participation in society based on six domains. A review on different models of disability and measurement method concluded that WG is a valid measure of function and consistent with ICF conceptual framework [7].

The prevalence of disability among population in Malaysia is expected to be high. Two major factors that contribute to this assumption are population profile and health status of its population. Malaysia is currently experiencing demographic transition with decreasing fertility rate and increasing life expectancy at birth, resulting in changes in population structure. Percentage of population aged 65 years and more is expected to increase significantly from 5.0% in 2010 to 14.5% in 2040, and Malaysia is expected to face population ageing in the year 2020, with 7.2% population within this age-group [8]. In addition, results from national surveys in Malaysia reported increasing prevalence of non-communicable diseases and its risk factors in Malaysia, with higher prevalence among older people [9–11]. In 2008, a study in one of the district in Malaysia found the prevalence of physical disability among older people as 19.5% [12].

Burden of disability increased with population ageing and increase in chronic health problems [4]. Analysis of the data in the United States revealed that even with similar prevalence of disability in 2005 (21.8%) as compared to 1999 (22.0%), the number of persons with disability increased due to population aging. The analysis also discovered that the top three causes of disability were arthritis, spine problem and heart problem [13]. Among the pre-retirement age, other than the musculoskeletal problems, disability was observed as associated with obesity and mental health [14].

In Malaysia, national data on disabilities are limited and different definitions were used in previous national surveys. National Health and Morbidity Survey (NHMS) 1996 reported 1.7% prevalence of disability; i.e. mobility and senses limitations [15], while NHMS in 2006 focused only on physical disability with a prevalence of 0.6% [16]. In both surveys, the disability was assessed for all ages. However, based on the analysis of WHS data in 2004, disability prevalence among adults in Malaysia was estimated as 3.1% [5].

Thus, a reliable data on disability based on tool that is consistent with ICF framework is vital for the policy makers and programme managers in the planning of inclusive programme for disability in Malaysia. A comprehensive data to relate disability with sociodemographic profiles and comorbidities will be more meaningful for the identification of target population to ensure equity in providing healthcare services. This study aims to examine the prevalence of disability among adults in Malaysia and its determinants, particularly the socioeconomic characteristics and comorbidities.

## Methods

### Study design and sampling

This study is part of the NHMS 2015, conducted by the Institute for Public Health, Ministry of Health Malaysia to supplement existing data and provide data for monitoring and evaluation of heath programmes implemented by the Ministry of Health. NHMS is conducted as regular survey since the first survey in 1986.

NHMS 2015 targeted Malaysian residents in the non-institutionalised living quarters in both urban and rural from all 13 states and 3 Federal Territories in Malaysia. Selection of samples was conducted by the Department of Statistics Malaysia using updated 2014 sampling frame. The frame was made up by 79,240 geographical areas known as Enumeration Blocks (EB) with 7.65 million living quarters (LQs). In average, each EB comprise of 80 to 120 LQ with 500 to 600 peoples.

In this survey, sample size was calculated based on single proportion formula for prevalence estimation. For disability module, based on 15% estimated prevalence [4], design effect of 2.0 and non-response of 35%, the sample size required for single strata analysis was 603. However, as this survey was consisted of several topics, the sample size for the whole survey was based on the biggest sample size required, with margin of error between 0.01 to 0.05, 95% confidence interval, adjusted for design effect of 1.5 to 2.0, and non-response rate of 35%. Based on the mentioned considerations, a total of 10,428 living quarters (LQs) from 869 Enumeration Blocks (EBs) were selected randomly. Allocation to the states was done as proportionate to the population size in each state and Federal Territories in Malaysia [17].

NHMS 2015 employed a two-stage stratified random sampling design to ensure national representativeness. Based on this design, states and Federal Territories were considered as Primary Stratum, while urban and rural areas within the states were Secondary Stratum. All states and Federal Territories were included in this survey, and within each state, selected number of EBs from urban and rural areas were randomly selected. Sample selection involved two stages; selection of EBs which were considered as Primary Sampling Unit and selection of LQs within the EBs which were considered as Secondary Sampling Unit. In this survey, out of 10,428 EBs, 536 EBs were selected from urban areas, with the remaining 333 EBs from rural areas, based on the proportion of Malaysian residents. In each EBs, a total of 12 LQs were randomly selected and all households and individuals within the selected LQs were invited to join this survey. Random selection of EBs and LQs were done by the Department of Statistics Malaysia.

Maps of those selected EBs with LQs were made available to the logistic teams prior to the data collection period. Based on the maps, logistic teams made preliminary visits to the selected LQs and identify occupied houses from those vacant or demolished. Residents of the houses were given pamphlets explaining about the survey. Based on the list of the occupied LQs, data collection teams made an appointment to visit for the interview. Minimum of at least 3 visits were made for each LQs. Consented individuals were invited to join the survey and respond to the questionnaires in NHMS 2015 including WG questionnaire.

### Questionnaire

The WG questionnaire was administered as a bilingual questionnaire; Bahasa Malaysia and English. The Bahasa Malaysia version of WG questionnaire was translated from English by two teams; language expert and content expert with fluency in English. The questionnaire was then back-translated to English by English-speaking experts with fluency in Bahasa Malaysia. The Bahasa Malaysia version was pilot tested in 30 individuals from three major ethnics in Malaysia; Malay, Chinese and Indians, and from both low and highly educated individuals. No problems were found in term of comprehension of the questionnaire content and wording. The questionnaire was then used in the survey and administered as a face-to-face interview using mobile devise. This module is only applicable to adults aged 18 years and above. The WG questionnaire consists of six functional domains; seeing, hearing, walking, cognition, self-care, and communication. The questionnaire starts with “The next questions ask about difficulties you may have doing certain activities because of a health problem”. For domain ‘seeing’ the question asked was “Do you have difficulty seeing, even if wearing glasses?”, followed by question “Do you have difficulty hearing, even if using a hearing aid?” for domain ‘hearing’, “Do you have difficulty walking or climbing steps?” for domain ‘walking’, “Do you have difficulty remembering or concentrating?” for domain ‘cognition’, “Do you have difficulty with self-care such as washing all over or dressing?” for the domain ‘self-care’ and “Using your usual (customary) language, do you have difficulty communicating, (for example understanding or being understood by others)?” for domain ‘communication’. Each question has four response categories: (1) No, no difficulty, (2) Yes, some difficulty, (3) Yes, a lot of difficulty and (4) Cannot do it at all.

Two major modules were covered in NHMS 2015: 1) household module; includes household characteristics and income, 2) individual module, which include sociodemographic and questionnaire on diabetes, hypertension, hypercholesterolemia, physical activity, smoking status, alcohol consumption, disability, and few other topics. Fasting blood sugar level was assessed using Cardiochek PA, a validated point-of care testing [18], while blood pressure measurement was performed using Omron Japan Model HEM-907, and Body Mass Index was assessed using SECA Portable Stadiometer 213 (SECA GmbH & Co. KG, Hamburg, Germany) and the TANITA Digital Weighing Scale HD 319 (TANITA Corp., Tokyo, Japan). For this paper, only data from those 18 years and above were extracted from the main database, including data on household income, individual characteristics, disability, non-communicable diseases and its risk factors, and healthcare utilisation.

### Variable definition

Disability was defined as having at least one of the six domains; seeing, hearing, walking, cognition, self-care, and communication, scored “a lot of difficulty or unable to do at all” or at least “some difficulty” scored in two domains. The cut-off was based on the definition used in Zambia [19].

Age was grouped into five categories; 18–30 years, 31–40 years, 41–50 years, 51–60 years and 60 years and more. In our survey, ethnicity was classified based on three major ethnic groups in Malaysia, namely Malay, Chinese and Indian, followed by ‘Other *Bumiputera*’ and ‘Others’. Other *Bumiputera* comprises of indigenous groups, local Sabahans and Sarawakians, while ‘Others’ were mostly foreigners, immigrants, both legal and illegal, residing in Malaysia.

Education levels were also based on the Malaysian education system. Respondents were considered as having no formal education if they had not attended any formal schooling. Those who had completed up to 6 years of primary school were considered as having attained primary education, while those who had completed 11 years of formal schooling were considered as completed secondary education. Respondents with diploma or higher qualifications were considered as having completed tertiary education.

Household income was calculated based on the pooled income of family members and divided into quintile; with the first quintile represent the poorest 20% and fifth quintile represent the richest 20%. Respondents were considered as having diabetes or hypercholesterolemia if they were told by the medical personnel as having diabetes or hypercholesterolemia prior to the survey or when fasting blood sugar was 6.1 mmol/L or more, or total cholesterol was 5.2 mmol/L or more, respectively. Respondents were considered as having hypertension when they were either told as having hypertension by medical personnel prior to the survey or resting blood pressure was 140/90 mmHg or more.

Mental health problem was screened using locally validated GHQ12, and respondents were categorised into having mental health problem if the total score was more than 3 [20]. GHQ12 is the shorter version of the original GHQ60, which was designed as self-administered screening test for detecting minor psychiatric disorders among respondents in community settings [21].

In this survey, respondents were considered as ‘current smoker’ if they admitted as currently smoke daily or occasionally any tobacco product, while ‘current drinker’ was defined as consumed alcohol in 12 months prior to the survey.

### Statistical analysis

Data was analysed using complex sample module in the IBM Statistical Package of Social Sciences (SPSS) for Windows version 23.0 (IBM Corp., Armonk, NY, USA), taking into consideration the complexity of the sampling design. The overall prevalence of disabilities and its estimated population affected were determined. Bivariate analysis was done to determine the association between sociodemographic and comorbidities variables with disabilities. Crude odds ratios were used to describe the strength of association between dependent and independent variables. Multivariable logistic regression model was fitted to determine the factors associated with disabilities. The independent variables that were included were locality, sex, ethnicity, household income, age group, education level, marital status, current smoker, current drinker, having mental health problem, physically inactive, Body Mass Index, diabetes, hypertension, and hypercholesterolemia. As the dependent variable was dichotomous, we used a logistic regression model to produce crude odds ratio as a measure of association. For the final model, FORWARD LR variable selection method was used to obtain significant variables. Only variables with *p*-value of less than 0.25 were included. The variables included were sex, age group, ethnicity, marital status, education level, having mental health problems, physically inactive, Body Mass Index, current smoker, current drinker and hypertension. The statistical significance of the individual regression coefficient was tested using Wald chi-square statistic. The adjusted OR, with the irrespective 95% confidence intervals (CIs), was then calculated. A p-value of less than 0.05 was considered significant. Model fit was tested by the Hosmer-Lemeshow statistic, which should be non-significant (*p* > 0.05). Additional analysis was then performed for older people 60 years and above, followed by similar steps, to identify the final model.

## Results

Out of 10,428 living quarters selected, 9433 were eligible, but only 8411 responded during the survey, with 89.2% response rate. From those responded household, 29,606 were successfully interviewed, with 19,959 adult aged 18 years and older eligible to answer this module.

By domain, our survey found that the prevalence of having at least “some difficulties” was highest in ‘seeing’; 16.8% (95% Confidence Interval, CI:15.9,17.8), followed by ‘walking’; 11.3% (95% CI:10.6, 12.0), ‘remembering’; 9.4% (95% CI:8.7, 10.1), and ‘hearing’, ‘communicating’ and ‘self-care’ with 5.5% (95% CI:5.0, 6.0), 3.4% (95% CI:3.1, 3.8), and 2.5% (95% CI:2.2, 2.8), respectively.

Further analysis was done to identify overall prevalence of disability among adults in Malaysia. Our study showed that the prevalence of having at least one domain scored as “some difficulty” was 25.9% with estimated more than 5 million adults in Malaysia affected, while 2.7%, estimated 553,865 adults, had at least one domain scored “a lot of difficulties” and 1.0% with estimated 195,924 adult population, scored at least one domain with “unable to do at all”(Table 1). Based on the definition of having at least one of the six domains scored “a lot of difficulty or unable to do at all” or at least “some difficulty” scored in two domains, the overall prevalence of disability among adults in Malaysia was 11.8% (95% CI: 11.15, 12.53) with an estimated 2.4 million persons affected.

**Table 1:** Prevalence of disability among adult population in Malaysia (*n* = 19,959)

Level of disability	Unweighted count	Estimated population	Prevalence % (95% CI)
At least one domain scored some difficulty	5,761	5,235,342	25.9 (24.78, 27.13)
At least one domain scored a lot of difficulty	659	553,865	2.7 (2.46, 3.06)
At least one domain scored unable to do at all	237	195,924	1.0 (0.82, 1.15)
At least one domain scored a lot of difficulty/unable to do, or, at least some difficulty scored in two domains	2802	2,387,716	11.8 (11.15, 12.53)

Table 2 shows sociodemographic profile of the adult respondents in NHMS 2015. Three-quarter of respondents reside in urban area, which is similar to the population distribution in Malaysia, with almost equal distribution by sex. By age-group, one-third of the respondents were between the age of 18 and 30 years old with only one-tenth older adults aged 61 years and above. Ethnicity was similar with ethnic distribution in Malaysia due to post-stratification adjustment. It was noted that more than one-third of our respondents were single or widow/er/divorcee, while more than half of our respondents attained minimum of secondary education level.

**Table 2:** Sociodemographic profiles of adults respondents, 18 years and above, NHMS 2015 (*n* = 19,959)

Variables		% (95%CI*)
Locality	Urban	75.8 (74.8, 76.8)
	Rural	24.2 (23.2, 25.2)
Sex	Male	51.6 (50.7, 52.4)
	Female	48.4 (47.6, 49.3)
Age-group (years)	18–30	35.4 (34.2, 36.5)
	31–40	22.3 (21.3, 23.4)
	41–50	17.3 (16.5, 18.1)
	51–60	13.1 (12.5, 13.9)
	≥61	11.9 (11.2, 12.6)
Ethnicity	Malay	49.6 (46.9, 52.3)
	Chinese	23.2 (20.9, 25.7)
	Indian	6.8 (5.8, 7.8)
	Other *Bumiputera*	10.9 (9.5, 12.6)
	Others	9.5 (8.0, 11.3)
Marital status	Single	33.2 (32.2, 34.4)
	Married	60.3 (59.2, 61.5)
	Widow/Widower/divorcee	6.4 (6.0, 6.9)
Household income	Poorest 20%	11.7 (10.9, 12.6)
	Poor 20%	17.0 (16.0, 18.1)
	Middle income	20.7 (19.5, 22.1)
	Rich 20%	21.9 (20.5, 23.3)
	Richest 20%	28.6 (26.9, 30.4)
Education level	No formal education /unclassified	7.4 (6.7, 8.1)
	Completed Primary Education	19.9 (18.9, 21.0)
	Completed Secondary Education	48.6 (47.3, 49.8)
	Completed Tertiary Education	24.1 (22.9, 25.4)
Diabetes mellitus		17.5 (16.6, 18.3)
Hypertension		30.3 (29.3, 31.2)
Hypercholesterolemia		47.7 (46.5, 48.9)
Body mass index	Underweight (BMI < 18.5 kg/m^2^)	6.7 (6.2, 7.3)
	Normal (BMI 18.5–24.9 kg/m^2^)	45.6 (44.5, 46.7)
	Overweight (25.0–29.9 kg/m^2^)	30.0 (29.1, 31.0)
	Obese (≥30.0 kg/m^2^)	17.7 (16.9, 18.5)
Physical inactivity		33.5 (32.4, 34.5)
Mental health problem		29.2 (27.9, 30.5)
Current smoker		22.8 (21.9,23.8)
Current drinker		8.0 (7.1, 9.1)

^*^ *CI* Confidence interval

Regarding the aspects of comorbidity, our study found that 17.5% of adults in Malaysia have diabetes, with 30.3% hypertensive and 47.7% have hypercholesterolemia. One-third of Malaysian adults were physically inactive, while 29.2% have mental health problems, 22.8% currently smoking and 8.0% were current drinkers.

Overall, prevalence of disability was found to be higher in rural area (crude odds ratio, OR: 1.42, 95% CI: 1.31, 1.53), and among females (crude OR: 1.13, 95% CI: 1.05, 1.23). The prevalence increased with age, with the highest prevalence among those 61 years and more, which is 16 times higher odds compared to those 18–30 years. The odds of having disability increased 2.5 times for other *Bumiputeras* compared to the Malays. With regards to marital status, the prevalence was twice higher among married adults, and 7 times higher among widow/widower compared to those who were single. Prevalence of disability was also higher among low household income compared to the richest 20% group. Those with lower education level were also shown to be at greater risk of disability compared to those with tertiary education level.

By comorbidities status, the odds of having disability were doubled among adults with diabetes, and almost triple among those with hypertension compared to those without the disease. The prevalence also higher among adults with hypercholesterolemia. There were positive associations between mental health problems (crude OR: 1.52, 95% CI: 1.34, 1.73) and physical inacitivity (Crude OR: 2.49, 95% CI: 2.29, 2.70) with disability. However, bivariate analysis showed no significant association between disability and BMI. Adults who currently smoke and drink were also less likely to report having a disability.

Further analysis using logistic regression revealed that population at risk of having disability in Malaysia were older people aged 61 years and above (adjusted OR, aOR: 12.86 (9.48, 17.45) compared to those 18–30 years, other *Bumiputeras* (aOR: 2.55, 95% CI: 2.06, 3.16), and those with low education. Those who were married were less likely to be disabled compared to those who were singles. After controlling for sociodemographic profiles, disability was found to be significantly higher among obese adults (aOR: 1.42, 95% CI: 1.20, 1.70) as compared to normal weight adults, adults with mental health problems (aOR: 1.68, 95% CI: 1.46, 1.94) as compared to those without mental health problems, and physically inactive adults (aOR: 1.43, 95% CI: 1.24, 1.64) as compared to those physically active (Table 3).

**Table 3:** Associated factors of disability among adult 18 years and above in Malaysia (n = 19,916)

	Variables	Prevalence (95% CI)	Crude OR (95% CI)	Wald	Adjusted OR^a^ (95% CI)	*p* value
Locality	Urban	10.81 (10.03,11.64)	R			
	Rural	15.08 (13.77, 16.49)	1.42 (1.31, 1.53)			
Sex	Male	10.78 (9.97, 11.66)	R			
	Female	12.93 (12.07, 13.84)	1.13 (1.05, 1.23)			
Age-group (years)	18–30	4.21 (3.55, 4.99)	R			
	31–40	5.31 (4.50, 6.26)	1.38 (1.13, 1.68)	12.34	1.72 (1.27, 2.32)	<0.001
	41–50	10.07 (8.84, 11.46)	2.68 (2.49, 3.20)	60.58	3.26 (2.42, 4.38)	<0.001
	51–60	19.24 (17.35, 21.28)	5.62 (4.77, 6.61)	152.34	6.39 (4.76, 8.58)	<0.001
	≥61	41.00 (38.57, 43.47)	16.04 (13.75, 18.72)	269.20	12.86 (9.48, 17.45)	<0.001
Ethnicity	Malay	10.82 (10.01, 11.69)	R		R	
	Chinese	12.59 (11.30, 14.01)	1.26 (1.13, 1.41)	0.02	0.99(0.81, 1.19)	0.88
	Indian	11.70 (9.80, 13.91)	1.05 (0.89, 1.23)	0.14	0.95 (0.73, 1.24)	0.71
	Other *Bumiputera*	17.95 (15.54, 20.65)	2.51 (2.01, 3.13)	74.15	2.55 (2.06, 3.16)	<0.001
	Others	8.45(6.34, 11.18)	0.71 (0.59, 0.87)	2.94	1.35 (0.96, 1.90)	0.09
Marital status	Single	6.48 (5.59, 7.51)	R		R	
	Married	11.75 (10.92, 12.62)	2.03 (1.79, 2.31)	10.69	0.65 (0.50, 0.84)	0.001
	Widow/Widower	33.99 (31.15, 36.96)	7.01 (6.03, 8.16)	1.02	0.85 (0.62, 1.17)	0.31
Household income	Poorest 20%	23.62 (21.38, 26.02)	3.54 (3.12, 4.02)			
	Poor 20%	14.67 (13.16, 16.33)	1.93 (1.70, 2.19)			
	Middle income	9.81 (8.68, 11.05)	1.34 (1.18, 1.53)			
	Rich 20%	9.21 (8.09, 10.47)	1.24 (1.08, 1.42)			
	Richest 20%	8.82 (7.82, 9.94)	R			
Education level	No formal education	31.76 (28.18, 35.57)	11.41 (9.60, 13.56)	34.69	2.75 (1.97, 3.86)	<0.001
	Completed Primary Education	22.52 (20.89, 24.23)	6.71 (5.75, 7.83)	42.54	2.10 (1.68, 2.62)	<0.001
	Completed Secondary Education	8.20 (7.46, 9.02)	1.98 (1.69, 2.32)	6.37	1.29 (1.06, 1.58)	0.01
	Completed Tertiary Education	4.34 (3.66, 5.14)	R		R	
Diabetes mellitus	Yes	20.57(18.9, 22.3)	2.22 (2.03, 2.42)			
	No	9.98 (9.32, 10.67)				
Hypertension	Yes	21.05 (19.7, 22.5)	2.89 (2.66, 3.13)			
	No	7.82 (7.22, 8.48)				
Hypercholesterolemia	Yes	14.03 (13.1, 15.0)	1.36 (1.25, 1.47)			
	No	9.82 (9.07, 10.63)				
Body mass index	Normal	10.18 (9.34, 11.09)	R		R	
	Underweight	10.03 (8.09, 12.38)	1.02 (0.85, 1.24)	0.01	0.98 (0.69, 1.40)	0.92
	Overweight	11.74(10.65, 12.92)	1.07 (0.97, 1.18)	0.12	1.03 (0.88, 1.21)	0.73
	Obese	12.19(10.88, 13.65)	1.10 (0.98, 1.24)	15.65	1.42 (1.20, 1.70)	<0.001
Mental health problems	Yes	9.99(8.75, 11.39)	1.52 (1.34, 1.73)	50.78	1.68 (1.46, 1.94)	<0.001
	No	7.03 (6.35, 7.78)			R	
Physical inactivity	Yes	17.77 (16.52, 19.08)	2.49 (2.29, 2.70)	24.38	1.43 (1.24, 1.64)	<0.001
	No	8.91 (8.20, 9.67)			R	
Current smoker	Yes	8.38(7.38, 9.49)	0.67 (0.60, 0.75)			
	No	12.92 (12.16, 13.71)				
Current drinker	Yes	8.19(6.46, 10.32)	0.65 (0.53, 0.80)			
	No	12.16 (11.44, 12.91)				

*CI* Confidence interval, *OR* Odds Ratio, ^a^adjusted for locality, sex, age group, ethnicity, marital status, household income, education level, Body Mass Index, physical inactivity, current smoker, current drinker, diabetes mellitus, hypertension, hypercholesterolemia, and mental health problem

As disability was significantly higher among those older people age 60 years and above, additional analysis among this age-group was done. Concerning different types of disabilities, the prevalence of difficulties in seeing, hearing, walking, remembering, self-care, and communicating were 38.9% (95% CI: 36.6, 41.3), 22.4% (95% CI: 20.5, 24.5), 40.6% (95% CI: 38.3, 42.9), 27.0% (95% CI: 25.0, 29.2), 10.3% (95% CI: 8.9, 11.8), and 12.2% (95% CI: 10.6, 13.9), respectively. A total of 58.3% older people had at least one domain scored moderately difficult and 40.0% (95% CI: 37.7, 42.3) were classified as disabled based on our definition. Logistic regression analysis revealed that among older people, prevalence of disability were significantly higher among those with no formal education; (aOR: 2.81, 95% CI: 1.60, 4.96) compared to those with tertiary education, having mental health problems (aOR: 1.68, 95% CI: 1.28, 2.20) compared to those without mental health problems, and physically inactive (aOR: 1.94, 95% CI: 1.52, 2.47) compared to those physically active, but lower among current smokers (Table 4).

**Table 4:** Associated factors of disability among older adult 60 years and above in Malaysia (*n* = 3794)

	Variables	Prevalence (95% CI)	Crude OR (95% CI)	Wald	Adjusted OR^a^ (95%CI)	*p* value
Locality	Urban	38.44 (35.60, 41.37)	R			
	Rural	44.04 (40.55, 47.60)	1.14 (1.00, 1.30)			
Sex	Male	36.53 (33.23, 39.96)	R			
	Female	43.35 (40.31, 46.45)	1.17 (1.03, 1.34)			
Ethnicity	Malay	39.76 (36.76, 42.84)	R			
	Chinese	36.09 (31.88, 40.51)	1.01 (0.85, 1.19)			
	Indian	40.74 (32.82, 49.16)	1.05 (0.80, 1.39)			
	Other *Bumiputera*	56.46 (49.04, 63.61)	2.05 (1.59, 2.66)			
	Others	39.89 (24.11, 58.09)	0.76 (0.42, 1.35)			
Marital status	Single	46.13 (31.71, 61.24)	R			
	Married	35.25 (32.51, 38.09)	0.75 (0.49, 1.17)			
	Widow/Widower	50.59 (46.61, 54.56)	1.33 (0.85, 2.08)			
Household income	Poorest 20%	43.57 (39.48, 47.74)	1.44 (1.16, 1.78)			
	Poor 20%	40.88 (36.41, 45.50)	1.18 (0.94, 1.48)			
	Middle income	37.63 (32.29, 43.29)	1.14 (0.89, 1.45)			
	Rich 20%	36.86 (30.96, 43.18)	1.10 (0.85, 1.42)			
	Richest 20%	37.50 (32.15, 43.18)	R			
Education level	No formal education	56.68 (51.89, 61.15)	3.90 (2.71, 5.61)	12.83	2.81 (1.60, 4.96)	<0.001
	Completed Primary Education	40.87 (37.83, 43.99)	2.06 (1.45, 2.92)	4.25	1.62 (1.02, 2.55)	0.28
	Completed Secondary Education	29.41 (25.35, 33.82)	1.28 (0.89, 1.85)	0.00	0.99 (0.62, 1.61)	0.99
	Completed Tertiary Education	18.73 (12.96, 26.30)	R		R	
Diabetes mellitus	Yes	43.33 (39.81, 46.82)	1.24 (1.08, 1.42)			
	No	38.02 (35.14, 40.99)	R			
Hypertension	Yes	42.34 (39.60,45.13)	1.21 (1.05, 1.40)			
	No	34.86 (31.13, 38.58)	R			
Hypercholesterolemia	Yes	39.87 (37.19,42.61)	1.20 (1.05, 1.38)			
	No		R			
Body mass index	Normal	36.48 (33.11, 40.00)	R			
	Underweight	55.00 (44.52, 65.07)	1.98 (1.46, 2.69)			
	Overweight	35.15 (31.15, 39.47)	0.90 (0.76, 1.05)			
	Obese	32.58 (27.78, 37.79)	0.92 (0.75, 1.13)			
Mental health problems	Yes	34.38 (27.91,4.48)	1.69 (1.32, 2.15)	14.01	1.68 (1.28, 2.20)	<0.001
	No	28.42 (24.74, 32.42)	R		R	
Physical inactivity	Yes	53.07 (49.71,56.40)	2.98 (2.60, 3.42)	28.98	1.94 (1.52, 2.47)	<0.001
	No	27.54 (24.77, 30.49)	R		R	
Current smoker	Yes	33.47 (28.07,39.35)	0.72 (0.59, 0.87)	4.65	0.68 (0.48, 0.97)	0.03
	No	40.98 (38.48, 43.52)	R		R	
Current drinker	Yes	23.00 (15.02,33.54)	0.39 (0.24, 0.64)			
	No	40.67 (38.35, 43.04)	R			

*CI* Confidence interval, *OR* Odds Ratio, ^a^adjusted for sex, ethnicity, educational level, marital status, Body Mass Index, mental health problem, current smoker, current drinker, physically inactive, hypertension

## Discussion

Based on the findings of this survey, the prevalence of disability among adults in Malaysia (11.8%) is comparable with the prevalence in Zambia (13.3%) in 2006 [19]. Both surveys used similar methodology and similar cut-off point of WG. A lower prevalence (9.1%) was found in a nation-wide Household Income and Expenditure Survey in Bangladesh using similar WG questionnaire, while a study in Bogra district in Bangladesh using Rapid Assessment of Disability (RAD) reported a prevalence of 10.5% in 2010 [22]. RAD comprise of WG statements with additional statements on psychological distress. Population-based surveys in North-West Cameroon and Telangana State, India, revealed disability prevalence as 10% and 12.2%, respectively [23]. In these two surveys, disability was defined as reported significant functional limitation using WG questionnaire, and/or having a moderate or severe clinical impairment, epilepsy or depression. A higher prevalence of disability (36.7%) among adults 18–65 years in Brazil was reported based on their nation-wide household survey in 2008 [24]. However, the Brazilian survey only focussed on physical mobility in performing various activities.

The prevalence of disability in Malaysia based on our survey (11.8%) is higher compared to the estimated prevalence in Malaysia in 2004 based on WHS, with similar definition of disability, which was only 3.1% [5]. Possible reasons for this sharp increased might be due to the increased in the prevalence of non-communicable diseases in Malaysia on top of increased proportion of elderly population in Malaysia [8, 9]. Both these factors may result in limitation of functions and probably more so without adequate intervention to halt the disabilities. This unmet need requires concerted efforts from whole spectrum of public health management; from early detection and prompt management to optimum rehabilitation. Currently, Ministry of Health Malaysia has implemented the Plan of Action on Health Care for Persons with Disabilities 2011–2020, aiming to improve and maintain the health of persons with disabilities (PWD) by providing equal opportunities for health care, to empower individuals, families and communities for self-care and development of support services for PWD, as well as to decrease the prevalence of disabilities through the provision of adequate medical rehabilitation services at all levels of care. At the same time, the Ministry of Health through the National Council for PWD, provides input and advice to the Government on the issues related to health and disabilities. Ministry of Health will highlight to the council areas where health programmes can be implemented through networking with agencies and non-government organisations, such as implementing programmes that aims in educating the society and raising their awareness, while at the same time provide training for healthcare providers and other agencies in providing services to PWDs.

The prevalence of disability from this survey might be underestimated as WG is a screening tool which only focus on limitation of function due to health problems. Mactaggart et al. found that more than one-third of people with disability, specifically impairment in vision or hearing, were identified clinically but did not report their functional limitation [23]. Sabariego et al. also commented the usage of a screener such as WG, which might missed-out population with mild to moderate disability but having difficulty in daily life [25].

Based on our survey, among adult population, disability was significantly higher among older people, ethnic minorities, those who are less educated, single, obese, physically inactive and having mental health problems. The positive relationship between disabilities and age was also seen in other studies [23, 26, 27]. Aging increased the risk of diseases, particularly chronic diseases, which lead to disabilities. Aging, even among those disease-free, resulted in increasing frailty, presented with decreased muscle strength and reduced cardiopulmonary fitness, which also resulted in functional limitation.

The association between disabilities and disadvantaged groups; ethnic minority and less educated people, were also observed in various studies [27–29] and also noted by WHO [30]. Those with disabilities may face barriers in accessing healthcare services and education, and vice versa. This vicious cycle may worsen their disabilities if not intervene at appropriate time. This unmet need should be focused upon in the planning of strategies for disabilities in Malaysia to ensure inclusiveness. The associations between disabilities with obesity and physically inactive are also well-known [31, 32]. The relationship between mental health and disabilities as seen in our study is also seen in a systematic review exploring the association between social relationship and mental health in persons with physical disabilities [33]. People with mental health problems may be less likely to seek treatment for early symptoms of physical illness due to issues such as cognitive impairment, social isolation, distrust of medical staff, and lack of social skills. There is possibility of discrimination issue whereby they often do not receive the same level of treatment as other people or they may be less likely to adhere to medical treatment regimens causing them more likely to become and remain disabled.

Our study revealed that older people who were at-risk for disabilities were those who have no formal education, having mental health problems and physically inactive. The relationship between low education level and disability also seen in a study in rural India [34]. Low education is closely related with low income status, which might become a barrier for health service utilisation. Our findings also in line with the German KORA-Age study [35] that found association between disabilities among older people with physical inactivity but not smoking. There is possibility that physical inacitivity among older people might be due to physical disability due to chronic diseases such as arthritis, which was not assessed in this study.

In our study, older people with with mental health problems were also at-risk for disabilities. Unfortunately we do not have the information on type of mental health problems faced by these older people. Possible causes of these mental health problems might be common psychiatric disorders among older people such as cognitive impairment and depression. Our findings concur with a large study involving older peoples from seven low and middle income countries that found dementia as the most important contributor to disability among elderly people [36].

### Strengths and limitations

The use of a large nationally representative sample in this survey provides a reliable and valid data for estimation of the disability prevalence among adults in Malaysia. This survey also used an internationally comparable validated tool which enables international comparison. One limitation of this study is the cross-sectional nature of this survey which prevents the identification of causal relationships between identified factors and disability. There is also possibility of reverse causality, for example; mental health problem such as depression might be the impact of disability and not the other way. The factors included in the study are also not extensive enough, as this study is not primarily focus on disability. Another limitation of this survey is the use of self-reported functional limitation without clinical confirmation.

## Conclusion

This survey provides reliable and internationally comparable data on disabilities in Malaysia. Our findings indicated that older age, ethnic minority, low education level and obese are significant risk factors for disability among adults in Malaysia. Among older people, disabilities are associated with low education, mental health problems, and physical inactivity. Findings from this survey highlighted the need for strengthening the health services targeting older people, those who are obese, physically inactive, with mental health problems and those in socially disadvantaged groups.

## Abbrreviations

EB: Enumeration Blocks
ICF: International Classification of Functioning, Disability and Health
LQs: Living quarters
NHMS: National Health and Morbidity Survey
WG: Washington Group on Disability Statistics
WHO: World Health Organization
WHS: World Health Survey
